# Asymptomatic 39 Weeks Abdominal Pregnancy – Video Report of a Case Occurred in Ivory Coast Resulting in a Live Birth

**DOI:** 10.34763/jmotherandchild.20232701.d-23-00001

**Published:** 2023-06-27

**Authors:** Pietro Iovenitti, Valentina Galiano, Andrea Finco, Francesca Tiberio, Okon Gerard, Emanuele Garzia, Privat Guie

**Affiliations:** Obstetrics and Gynecology Unit, ASST Santi Paolo e Carlo, Ospedale San Carlo Borromeo, Milano, Italy; Obstetrics and Gynecology Unit, ASST Melegnano-Martesana, Ospedale Santa Maria delle Stelle, Melzo, Italy; Obstetrics and Gynecology Department, CHU de Treichville University Hospital, Abidjan, Ivory Coast; Reproductive Medicine Unit, ASST Santi Paolo e Carlo, Ospedale San Paolo, Milano, Italy

**Keywords:** abdominal pregnancy, ectopic pregnancy, omental placental implantation, live birth abdominal pregnancy, women's health

## Abstract

**Background:**

Despite the current advances in antenatal care and imaging methodologies in obstetrics, cases of advanced abdominal pregnancies are still reported, mostly in low- and middle-income countries where frequently only a few perinatal checks are performed and where these methodologies are sometimes not adopted in obstetrical outpatient settings.

**Case presentation:**

We report the video of a case of a 20-year-old I gravida Ivorian patient, sent to CHU de T reichville in Abidjan, Ivory Coast, for management of abdominal 39 weeks pregnancy after routine antenatal care. She was asymptomatic with a live foetus in transverse lie position. The anamnesis revealed four prenatal checks without ultrasound evaluation, the first one at 24 weeks of pregnancy. Emergency median longitudinal sub-umbilical laparotomy incision was performed. Foetal extraction was realized by transplacental incision due to omental placental implantation. A live female baby weighting 3350 grams was delivered, presenting bilateral clubfeet and an enlarged neck. The release of the adherent placenta required a partial omentectomy and left adnexectomy and was carefully removed following active bleeding from its detached margins. The newborn died of respiratory distress on the first day after birth. No autopsy was performed. Postoperative morbidity for the woman was minimal and she was discharged on the seventh post-operative day in good general condition.

**Conclusion:**

Abdominal pregnancies with a normal live foetus at such an advanced gestational age are extremely rare, and there are no available videos in the extant literature of the surgical procedure performed. Standardization of treatment principles, pre-operative preparation with imaging techniques (MRI, embolization of placental vessels) and adequately equipped and staffed neonatal units are necessary to optimize the foetus-maternal outcomes.

## Introduction

Despite advances in diagnostic imaging and focused antenatal care, cases of undiagnosed abdominal pregnancies at term are still reported in obstetric practice, mostly in low- and middle-income countries [1, 2]. They are frequently missed in routine antenatal care in resource-limited settings or for few/no obstetric check-ups of the pregnant women and delayed diagnosis is usually associated with poor foetal and maternal outcomes, including death [3]. An abdominal pregnancy is a rare and highly morbid typology of extrauterine pregnancy that requires skilled management. It accounts for approximately 1% of ectopic pregnancies and ectopic pregnancies account for 1–2% of all pregnancies [4]. Asymptomatic presentation late in pregnancy is unusual and rarely results in a live birth at term with no evidence of growth restriction or preeclampsia in the mother, despite the unfavorable implantation site. In addition, abdominal pregnancies are associated with a high risk of surgical complications and postoperative morbidity [5].

### Case Report

We report a case of a 20-year-old I gravida Ivorian patient, sent to CHU de Treichville in Abidjan, Ivory Coast, for management of abdominal 39 weeks pregnancy after a routine antenatal care consultation and ultrasound at term of gestation. She was asymptomatic with a live foetus in transverse lie position. The anamnesis revealed four prenatal checks without ultrasound evaluation, the first one at 24 weeks of pregnancy.

The diagnosis of abdominal pregnancy was confirmed at the CHU after a new obstetrical evaluation and a TA ultrasound. TV ultrasound showed a non-pregnant uterus regular in morphology and volumetry. Antenatal nonstress test (NST) revealed only a persistent tachycardia with baseline fetal heart rate between 160–180 bpm. Emergency median longitudinal sub-umbilical laparotomy enlarged to para-umbilical incision was performed. Foetal extraction was realized by trans-placental incision due to abdominal placental implantation. A live female baby weighting 3350 grams and measuring 51 cm was delivered, with an Apgar score of 3-5-5 at the 1st, the 5th and the 10th minute respectively and presenting bilateral clubfeet and enlarged neck. It was not possible to perform cord pH to evaluate signs of neonatal acidemia. No following imaging investigations were performed on the newborn.

The placenta adhered to the epiploon, left ovary and salpinx and its release required a partial omentectomy and left adnexectomy. The placenta was carefully removed following active bleeding from its detached margins. The uterus appeared normal in morphology, with a size of 12 gestational weeks. The patient was transfused with a total of 3 units of red cell concentrate (1500 ml in total) during and after surgery. The newborn died on the first day after birth of respiratory distress. No autopsy was performed. Postoperative morbidity for the woman was minimal with transient paralytic ileus on the second post-operative day. Her recovery was otherwise uneventful and she was discharged on the seventh postoperative day in good general condition. Follow up evaluations of the mother at 2 weeks, 6 weeks, and 6 months postpartum were uneventful.

## Discussion and Conclusions

Abdominal pregnancy with a normal live foetus at such an advanced gestational age is rare. Our survey of the literature revealed fewer than 10 postdated reported cases of abdominal pregnancy [1, 6], mostly in the context of under-equipped health centres. In no case is there an available video of the surgical procedure performed. The higher incidence of abdominal pregnancy in developing countries is associated with low socioeconomic status, pelvic infections and substandard or poor obstetrical care—all are more prevalent in these countries.

In this specific case, the patient did not perform any ultrasound during the poor prenatal check-ups she did, which meant that the diagnosis of abdominal pregnancy was made late in the 39th week of gestation.

Both maternal and foetal mortality rates in such cases are high, as well as the malformation rate among the affected foetus [3]. In a more fortunate health centre, particularly one with good prenatal diagnostic facilities, the planning of management, delivery and clinical outcome may well have been different despite the late presentation at 39 weeks with the benefit of a multi-disciplinary team including geneticists, neonatal intensivists, and vascular and GI surgeons complementing the obstetric teams.

Treatment is surgery. Immediate termination of pregnancy can be offered if the diagnosis is made before 20 weeks of gestation [7]. Patients diagnosed with advanced abdominal pregnancies with stable conditions can be monitored under close surveillance and surgical delivery can be scheduled at 34 weeks of gestation after lung maturity is achieved [7, 8]. Although removal of the placenta carries a higher risk of haemorrhage, a partially detached placenta can be delivered with minimal morbidity and a good maternal outcome.

Given the documented low survival rates of neonates in such cases, neonatal units must be adequately equipped and staffed to support them. The issue of removing the placenta during the operation is controversial. Treatment of the remaining placenta with methotrexate has recently been abandoned in favor of the clinical and instrumental monitoring of the patient in the short and long term. Where available, interventional radiology procedures allowing for preoperative embolization of placental vessels are helpful in preventing severe intraoperative blood loss [9] and imaging such as MRI may help clearly demonstrate placental attachments to the bowel, omentum and other intra-abdominal organs prior to surgery.

These pre-operative procedures would make the surgeon aware of what to expect at surgery and reduce the associated maternal morbidity and mortality.

## Supplementary Material

Supplementary Material Details

Supplementary Material Details
