# Supplementary material for: Asymptomatic 39 Weeks Abdominal Pregnancy – Video Report of a Case Occurred in Ivory Coast Resulting in a Live Birth
Source: J Mother Child. 2023 Jun 27;27(1):30–2. doi: 10.34763/jmotherandchild.20232701.d-23-00001 (PMC10298481; doi:10.34763/jmotherandchild.20232701.d-23-00001)

**Figures**

**Figure 1.** The appearance of the non-pregnant uterus at the opening of the abdomen

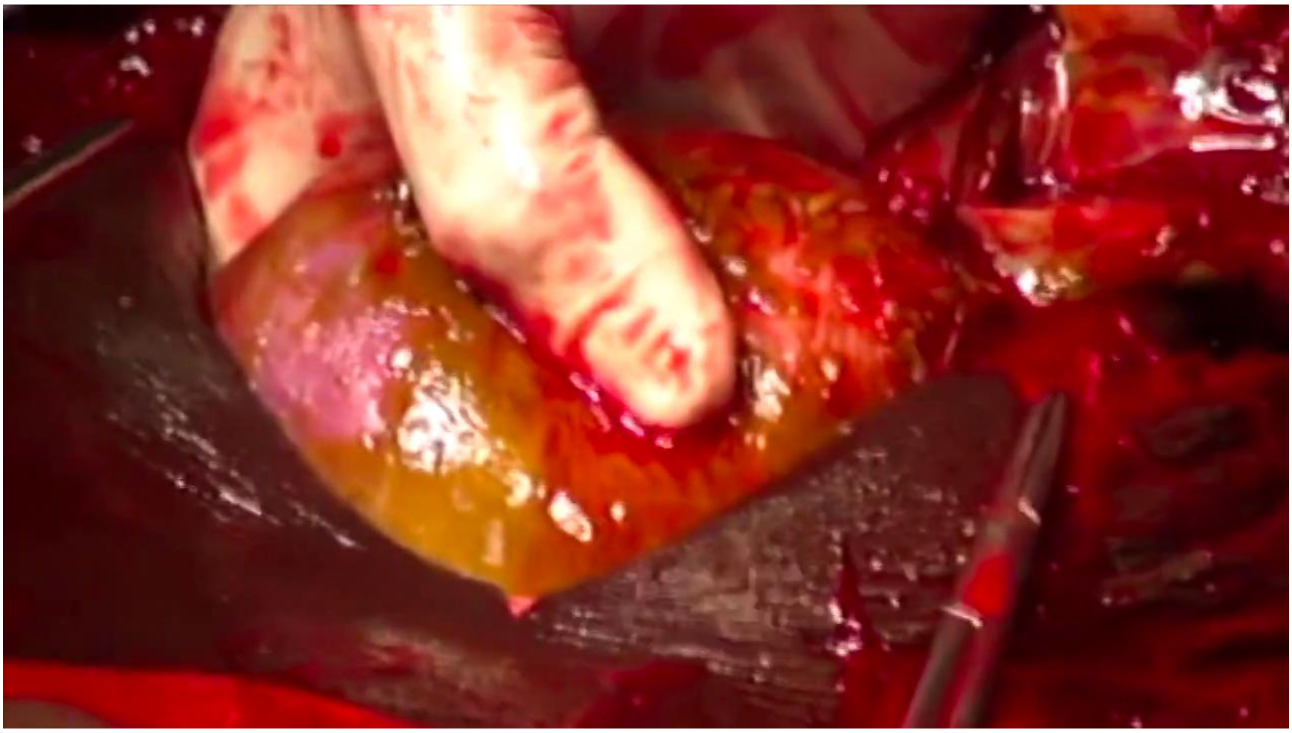

**Figure 2.** The appearance of the extensively adherent placenta after the removal from abdominal structures (epiploon, left salpinx and ovary)

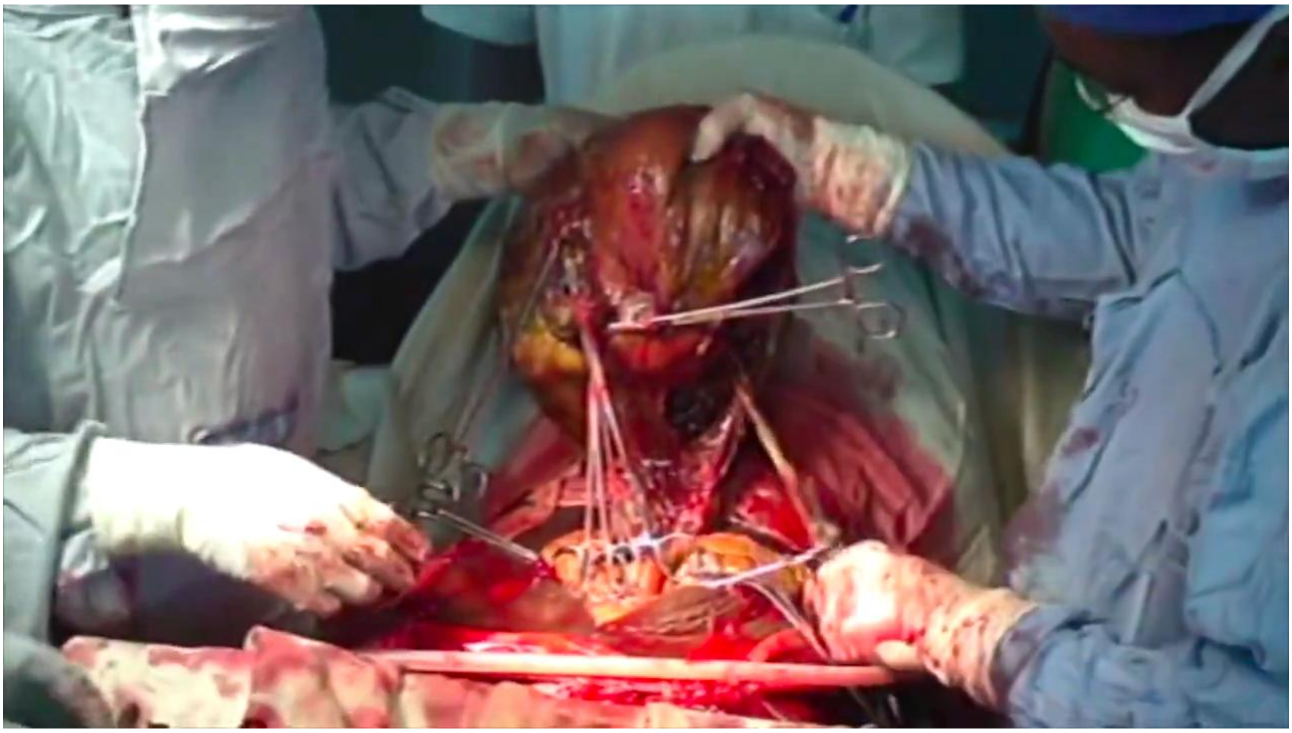

**Figure 3.** Newborn after extraction

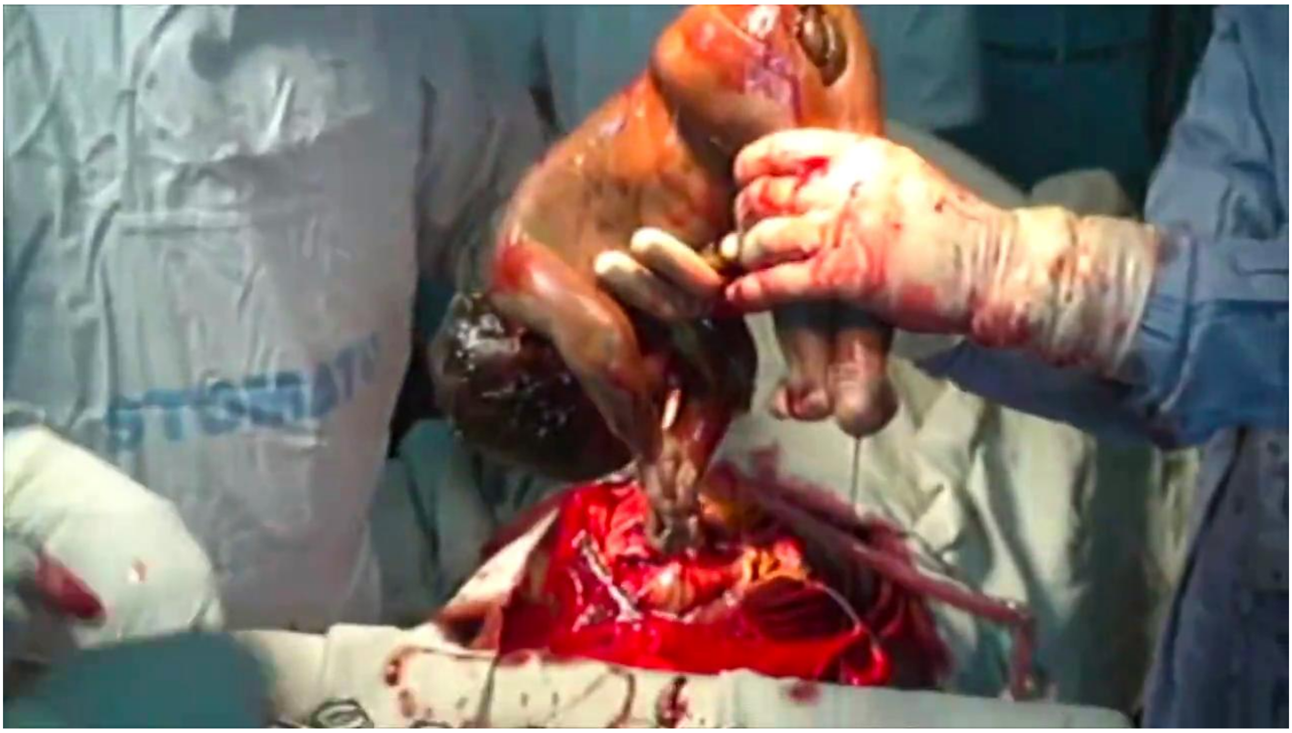

Supplement: Supplementary file 2 — Supplementary Material Details [file jmotherandchild.20232701.d-23-00001_sm2.pdf]
